# Terahertz Metamaterial Absorber and Equivalent Circuit Model for Refractive Index Sensing

**DOI:** 10.3390/ma18040765

**Published:** 2025-02-10

**Authors:** Zhengxiong Lu, Peixuan Li, Chuanwei Zhang, Shuaitian Li, Ruibo Chen, Ziliang Zhou, Xiaojun Huang

**Affiliations:** 1 School of Safety Science and Engineering, Xi’an University of Science and Technology, Xi’an 710054, China; luxust@163.com (Z.L.); pxli@xust.edu.cn (P.L.); 2 School of Mechanical Engineering, Xi’an University of Science and Technology, Xi’an 710054, China; zhangcw@xust.edu.cn (C.Z.); gjf1031@126.com (S.L.); 3 College of Communication and Information Engineering, Xi’an University of Science and Technology, Xi’an 710054, China; 24207223135@stu.xust.edu.cn (R.C.); 24251111412@stu.xidian.edu.cn (Z.Z.)

**Keywords:** terahertz, metamaterial absorber, equivalent circuit, refracting index sensing

## Abstract

As a kind of important functional device, terahertz metamaterial absorbers (TMA) have been focused on by many researchers for their capacity to absorb electromagnetic waves and wide application fields. In this work, we designed a terahertz metamaterial absorber with narrow-band absorption for refractive index sensing, which consisted of a circular metal ring resonator and a square metal ring resonator. The simulation results show that the absorptivity of the proposed TMA reached over 68.8% and 93.27% at 1.926 and 4.413 THz, respectively. Moreover, the absorption mechanism was studied through the electromagnetic field energy distribution, and the influence of structural parameters on absorption performance was exhibited. In refractive index sensing, a high sensitivity (S) of 2.537 THz/RIU (refractive index unit, RIU) was achieved by utilizing the coupling of ring resonators. The maximal quality factor (Q-factor) and figure of merit (FOM) of the TMA were 234.73 and 147.67 RIU^−1^, respectively. Additionally, we established an RLC equivalent circuit model (ECM) for the TMA, and we further illustrated the performance of the TMA in refractive index sensing through fitting the sensitivity based on the ECM to the sensitivity of the TMA. Our study exhibits the considerable potential application for the field of terahertz sensing, and the ECM for refractive index sensing will be helpful for continual investigation.

## 1. Introduction

Metamaterials have attracted the extensive attention of researchers for decades in wide application fields due to their many unnatural properties, like negative dielectric constant [1], negative permeability [2], negative refractive index [3], epsilon-near-zero [4], etc. Since Landy [5] designed the first metamaterial absorber (MA) with perfect absorption in 2008, MAs have played an important role in metamaterial devices. Researchers have proposed many designs for MAs covering wave bands such as microwave [6,7], terahertz [8,9], infrared [9,10,11], visible [12,13], ultraviolet [14,15], and multiband [16,17]. Furthermore, application fields like stealth [18,19], detectors [20,21], transceiver systems [22], imagers [21,23], thermal emitters [24,25], etc., have also been explored.

In 2010, Na Liu et al. presented a plasmonic sensor based on infrared perfect MA [26] whose performance parameter FOM* was nearly four times that of sensors based on plasmonic gold nanorods. Thus, the application of MAs in the sensing field emerged and rapidly became a hot topic. After that, an increasing number of MAs were reported for sensing applications in many fields, such as microelectromechanical system sensors [27,28], temperature sensors [29,30], humidity sensors [31,32], pressure sensors [33,34], and refractive index sensors [35,36,37].

With the rapidly expanding application fields, the research area of terahertz sensing has become significant for terahertz technology. Thus, TMAs for sensing have thrived and many methods have been proposed to improve the sensing performance of TMAs. Longqing Cong et al. presented two different TMAs with experimentally demonstrated higher sensitivity compared with planar metasurfaces [38], which consist of corresponding single metal pattern layers on the same silicon substrate. Xin Hu et al. suggested a TMA integrated microfluidic sensor with high sensitivity [39], as the concentrated electromagnetic field energy in etched dielectric spacers between two parallel metal structures interacts sufficiently with the detecting analytes. Hong Zhou et al. proposed a TMA with a bilayer cross-shaped plate–hole structure, achieving high sensitivity due to the fact that the positions of hot-spots overlap with the analyte spatially [40]. Based on previous works, an increasing number of TMAs with high sensitivity in refractive index sensing have been proposed [41,42,43,44,45,46]. Nonetheless, there is a margin for further enhancing the sensing performance, and a model for sensing is necessary.

In this paper, we designed a terahertz metamaterial absorber with narrow-band absorption for refractive index sensing. The structure of the suggested TMA is metal–dielectric–metal, with three layers consisting of gold and polyimide materials, and the top gold pattern layer consists of a circular and a square ring resonator. The full-wave simulation was carried out using CST microwave studio to determine the absorption performance of the proposed TMA. The simulation results show that the narrowband absorptivity of the proposed TMA reached 68.8% and 93.27% at 1.926 and 4.413 THz, respectively. The absorption mechanism was investigated through the electromagnetic energy distribution. Moreover, the influence of geometric parameters on the absorption performance was also studied. Then, an RLC ECM was established for the proposed TMA, the results of which showed good agreement with the full-wave simulation results. In refractive index sensing, the suggested TMA achieves a high sensitivity of 2.537 THz/RIU by utilizing the coupling of ring resonators, and the Q-factor and FOM of the TMA are 234.73 and 147.67 RIU^−1^, respectively. The refractive index sensing performance was further investigated through the ECM, whose sensitivity fit the corresponding result from CST well. The presented TMA demonstrates the considerable potential application for the field of terahertz sensing, and the ECM for refractive index sensing will be helpful for continual investigation.

## 2. Design and Simulation

**Figure 1:**
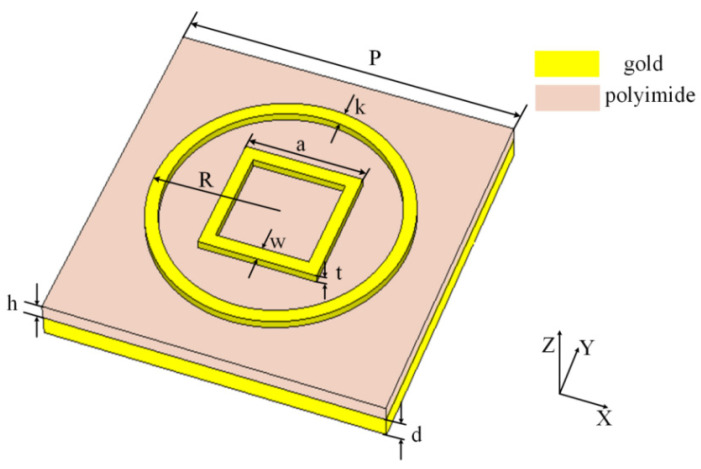
Schematic of the unit cell structure.

The unit cell structure of the proposed TMA is shown in Figure 1. It is a three-layer structure composed of a gold substrate, dielectric spacer, and gold pattern layer. In the top layer, the gold pattern consists of a circular and a square metal ring resonator. The material of the dielectric spacer is polyimide with a relative permittivity of ε_r_ = 3.5, and the loss tangent of the polyimide is 0.0027. The conductivity of gold is 4.56 × 10^7^ S/m. Furthermore, the detailed geometric parameters of the unit cell are given in Table 1.

In terms of simulation, we carried out all simulations by using the commercial software CST microwave studio 2022 based on the finite element method. The frequency domain analyzer was set as the simulation solver to determine the absorption curves of the TMA using simulation and calculation. It is noteworthy that the mesh type was tetrahedral. Furthermore, in directions of x and y, we set the conditions of the boundary as the unit cell. In the direction of Zmax, we set the boundary condition as open-add space. In the Z_min_ direction, the boundary condition was set as grounding (Et = 0).

## 3. Results and Discussion

### 3.1. Narrow-Band Terahertz Metamaterial Absorber

A = 1 − R − T = 1 − |S_11_|^2^ − |S_21_|^2^ was the equation used to calculate the absorptivity of the proposed TMA, in which S_11_ and S_21_ represent the coefficients of reflection and transmission, respectively. Thus, R and T corresponded to the reflection ratio and transmission ratio, respectively. The equation can be further simplified as A = 1 − R = 1 − |S_11_|^2^, since the existence of a gold substrate with sufficient thickness (much thicker than skin depth in terahertz) effectively prevents the electromagnetic wave from transmission.

**Figure 2:**
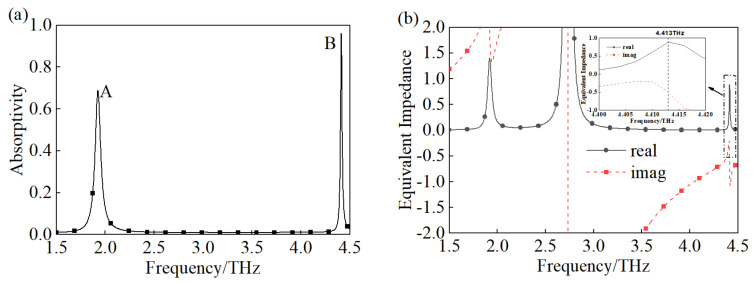
(**a**) The absorptivity of proposed TMA, (**b**) the equivalent impedance of the proposed TMA.

With the vertical incident of the terahertz TE wave, the absorptivity of the proposed TMA is shown in Figure 2a. There are two resonate peaks at 1.926 and 4.413 THz with absorptivity of 68.8% and 93.27%, respectively. We defined the resonate peaks at 1.926 and 4.413 THz as peaks A and B to simplify the description, respectively. The full width at half maxima (FWHM) of the resonate peak B was 0.0188 THz. Therefore, the proposed structure achieved narrow-band absorption. In addition, the equivalent impedance of the proposed TMA is demonstrated in Figure 2b, which was calculated by the equation $Z=\sqrt{(1+S_{11}{)}^{2}/(1-S_{11}{)}^{2}}$, and the equation was simplified due to the zero-transmission caused by the existence of the gold substrate. The impedance here is the normalized impedance. According to the impedance matching theory, the perfect absorption is generated when the equivalent impedance of TMA matches the impedance of the free space absolutely, which means that the real and imaginary part values of equivalent impedance are one and zero, respectively. As shown, the values of impedance at 1.926 THz do not matching the impedance of the free space well, so the absorptivity at 1.926 THz is only 68.8%. The equivalent impedance at 4.413 THz is a good match with the impedance of the free space, which leads to a high absorptivity of 93.27%.

**Figure 3:**
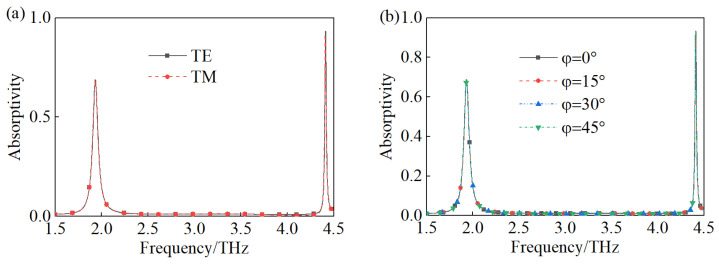
The performance of the TMA when (**a**) TE and TM waves irradiate vertically, (**b**) the polarization angle increases from 0° to 45°.

The results shown in Figure 3 demonstrate the polarization character of the proposed TMA. In Figure 3a, there are the absorption curves obtained from vertically irradiating TE and TM waves, respectively. Obviously, the two absorption curves almost coincide absolutely, which is due to the central rotational symmetry structure of the TMA. Figure 3b shows the absorption performance of TMA with a vertical incident of the TE wave as the polarization angle increased from 0° to 45° with a step width of 15°. It is apparent that the performance of TMA barely changed with the polarization angle increasing for the same reason as the structure.

**Figure 4:**
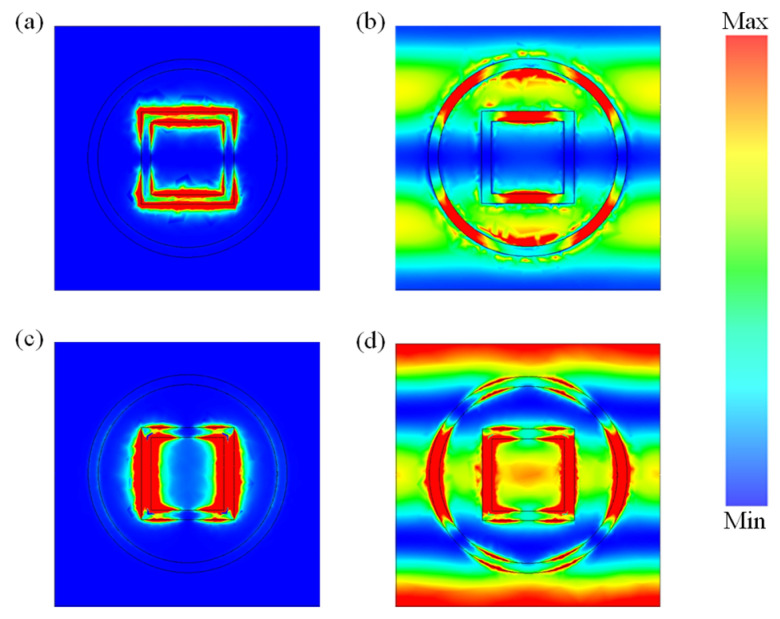
The energy distribution of (**a**) electric field at 1.926 THz, (**b**) electric field at 4.413 THz, (**c**) magnetic field at 1.926 THz, (**d**) magnetic field at 4.413 THz.

To illustrate the mechanism of the proposed TMA, we investigated the energy distribution of the electric and magnetic fields displayed in Figure 4. The results in Figure 4a,c are the electric and magnetic fields’ energy distributions of peak A. As shown, the electric field energy surrounded the two horizontal arms of the square ring. There were also large amounts of electric field energy in the corresponding space of dielectric layer below the arms of the square ring, which was due to the Fabry–Pérot (F-P) cavity, while the magnetic energy was distributed mainly in the vertical arms of the square ring at a similar location, and a slight amount of magnetic field energy covered the space between the square and circular rings upon the dielectric layer. It is clear that the absorption of peak A was generated mainly by the resonate of the square ring, and the faint coupling of ring resonators also provided a small contribution. The electric and magnetic fields’ energy distributions of peak B are exhibited in Figure 4b,d. As shown, the main electric field energy was distributed in the circular ring resonator, the square ring resonator, and the corresponding space of the dielectric layer below two metal ring resonators horizontally. The rest of the electric field energy covered the space between the circular ring and the square ring and the outside space of the circular ring. The phenomenon was caused by the coupling of metal ring resonators. The magnetic field energy was distributed at a position akin to the electric energy distribution, but in the vertical direction. Thus, the absorption of peak B was generated by the resonance and coupling of metal rings.

**Figure 5:**
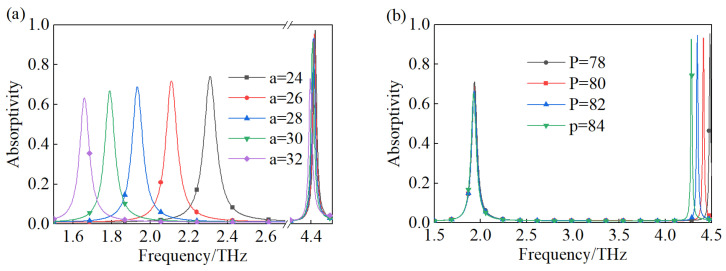
The performance of the proposed TMA with (**a**) variation in the square ring arm length, (**b**) variation in the period.

As a metamaterial device, the performance of the suggested TMA depends on its structural geometric parameters, and we studied several structural geometric parameters and how they influence the absorption capacity of the TMA. As shown in Figure 5a, with the value of square ring arm length ‘a’ increasing, the resonate frequency and absorptivity of both peaks A and B show red shifts and decline, respectively. For peak A, the absorption majorly results from the resonate of the square ring, so the dimension of the square ring corresponds to the operation frequency, and an obvious change can be observed. On the other hand, for peak B, the red shift of the resonate frequency and the reduction in absorptivity are caused by the variation in the coupling condition with the increasing arm length of the square ring. In Figure 5b, the influence of the period parameter ‘P’ on the performance of the proposed TMA is exhibited. When the period parameter rises, there is a slight influence on the absorptivity of peak A, while for peak B, the evident frequency red shift and the fluctuation of absorptivity are shown. This phenomenon is due to the fact that the operating frequency of peak A mainly depends on the dimensions of the square ring, while the operating frequency of peak B mainly relies on the period. Furthermore, the variate of the period parameter also changes the coupling situation between adjacent unit cells.

**Figure 6:**
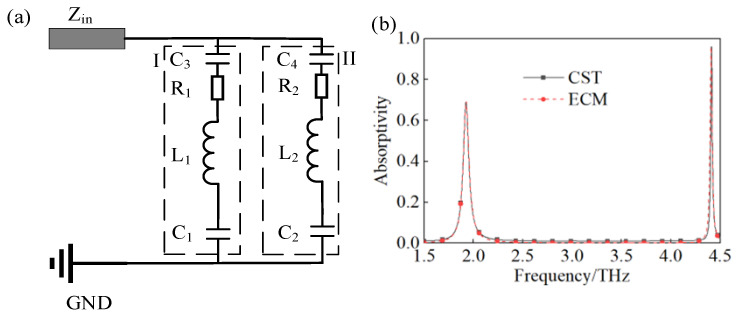
(**a**) RLC ECM of the presented TMA, (**b**) absorptivity obtained by CST and ECM.

To explain the results of the full-wave simulation conducted in CST, we investigated the RLC equivalent circuit model of the suggested TMA. As shown in Figure 6a, the RLC equivalent circuit model consists of parallel branches I and II, and every branch is an RLC series circuit. The branch circuits I and II represent the square and circular ring resonators with the corresponding F-P cavity. In the ECM, Zin is the impedance of the free space. R1, L1, and C1 in branch circuit I represent the equivalent impedance of the gold square ring and dielectric layer, the equivalent inductance of the gold square ring, and the coupling capacitance between the gold square ring and gold substrate, and their values are 109 Ω, 1.118 nH, and 6.2 × 10^−6^ pF, respectively. In branch circuits II, R2, L2, and C2 are the corresponding parameters of the circular gold ring, with values of 251 Ω, 4.016 nH, and 0.92 × 10^−6^ pF. It is notable that C3 and C4, with values of 4 × 10^−4^ pF and 0.5 × 10^−6^ pF, represent the coupling capacitance between the ring resonators, such as the circular ring with the square ring in a unit cell and circular rings in the adjacent unit cells. Figure 6b displays the absorption curves of the full-wave simulation using CST and ECM simulation in ADS. The good agreement of the two results indicates the effectiveness of the presented ECM.

### 3.2. Refractive Index Sensing

In this step, we investigated the refractive index sensing performance of the proposed TMA for the surrounding environment. There are three parameters, including S, Q-factor and FOM, characterizing the sensing performance. Their equations are as follows:

(1) $$S=\frac{\Delta f}{\Delta n}$$

(2) $$Q=\frac{f}{FWHM}$$

(3) $$FOM=\frac{S}{FWHM}$$

(4) $$f=\frac{1}{2\pi \sqrt{L(C+C_{C})}}$$

where Δ*f* is the frequency shift of the resonate peak as the surrounding environment’s refractive index Δ*n* changes, and f represents the resonate frequency.

**Figure 7:**
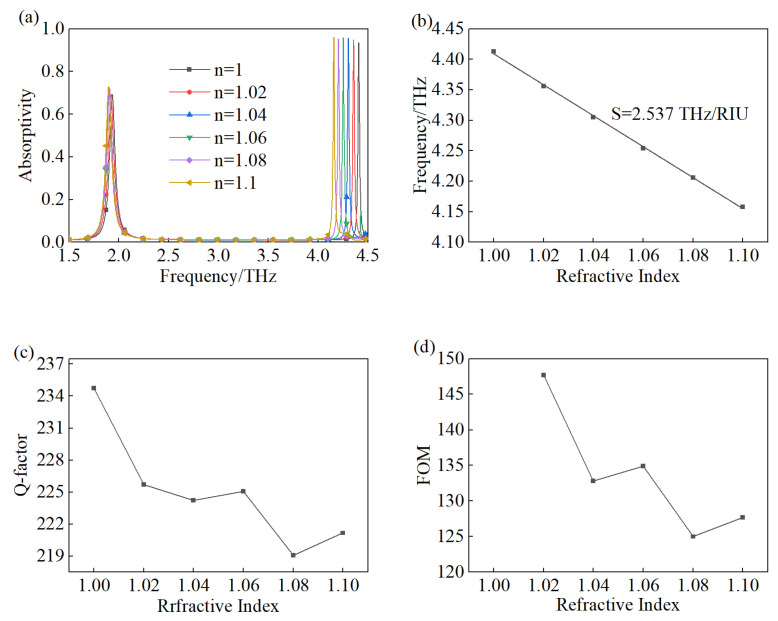
Refractive index sensing performance of proposed TMA: (**a**) absorptivity for the variety of refractive index, (**b**) frequency and sensitivity, (**c**) Q-factor, (**d**) FOM.

As shown in Figure 7a, we simulated the absorptivity of the suggested TMA when the environmental refractive index was set from *n* = 1 to *n* = 1.1, with a step width of 0.02. For peak A, slight variations in absorptivity and frequency were observed. In other words, peak A was not sensitive enough to match the demand of refractive index sensing. Meanwhile, there were significant frequency red shifts and little fluctuation in the absorptivity of peak B with the rising value of the refractive index, demonstrating that peak B was much more sensitive than peak A. Hence, we merely studied the performance of peak B for refractive index sensing. In Figure 7b, the six points correspond to the frequency of peak B with different refractive indexes, and the highest sensitivity can achieve 2.85 THz/RIU, as calculated by Equation (1), when the refractive index changes from *n* = 1 to *n* = 1.02. Furthermore, the line linearly fits peak B’s frequency, whose function is f = −2.537n + 6.946 with an R2 of 0.999, so that the sensitivity of the proposed TMA is a whopping 2.537 THz/RIU. In Figure 7c,d, the Q-factor and FOM are displayed, and the maximum Q-factor reaches 234.73, as calculated by Equation (2), when *n* = 1, while the largest FOM can be obtained at *n* = 1.02 with the value of 147.67.

**Figure 8:**
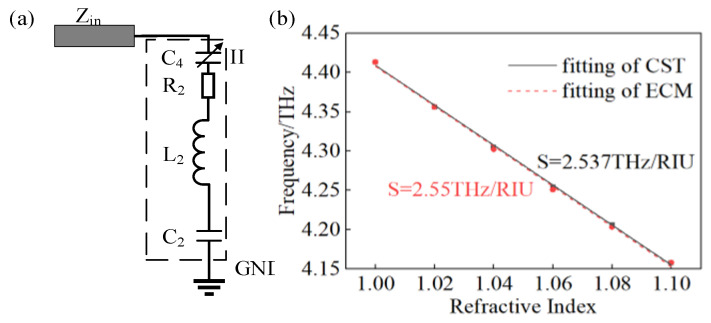
(**a**) Modified ECM for refractive sensing, (**b**) sensitivity comparison of results from CST and ECM.

According to Equation (4), when the environmental refractive index is increasing, there is a red shift of the resonate frequency, which agrees with the phenomenon shown in Figure 7. Therefore, the modified branch circuit II of ECM for refractive index sensing is exhibited in Figure 8a. The symbol of coupling capacitance C4 is changed to a symbol of variable capacitance to indicate that the value of C4 has square variation corresponding to the varying value of the environmental refractive index. As shown in Figure 8b, the frequency points of full-wave simulation in CST and ECM simulation in ADS nearly coincide completely, and there is also only a slight disparity in the linear fitting lines. The fitting function of the result gained from ECM is f = −2.55n + 6.958 with R2 of 0.998, which means that the sensitivity of ECM is 2.55 THz/RIU, and compared with the sensitivity of the proposed TMA, whose value is 2.537 THz/RIU, there is a deviation of merely 0.51%.

Finally, a comparison between our study and previous works is given in Table 2 to demonstrate the high performance of our TMA in refractive index sensing.

## 4. Conclusions

In summary, we proposed a TMA with narrow-band absorption for refractive index sensing, which consists of a circular and a square metal ring resonator. The structure of the TMA is metal–dielectric–metal, and the corresponding metal and dielectric materials are gold and polyimide. The simulation results demonstrate that the TMA can obtain absorptivity values over 68.8% and 93.27% at 1.926 and 4.413 THz. Meanwhile, the polarization-insensitive character of the TMA is due to the central rotational symmetry structure. Then, we established an RLC-equivalent circuit model for the TMA, the absorption curve of which matched the simulation results from CST well. Moreover, we studied the refractive index sensing performance of the TMA. The high sensitivity of 2.537 THz/RIU was due to the fact that the coupling of the ring resonators concentrated the electromagnetic energy in the space between the ring resonators, which was sensitive to the refractive index variation of the surrounding environment. The Q-factor and FOM were 234.7 and 147.67 RIU^−1^, respectively. We further investigated the refractive index sensing performance of the TMA based on the equivalent circuit model. The sensitivity of the TMA simulated in CST was 2.537 THz/RIU, and the corresponding result of ECM was 2.55 THz/RIU, which shows a good fit. Additionally, the high refractive sensing performance of our TMA was illustrated through a comparison with previous works. Thus, our TMA exhibited considerable potential for application in the field of terahertz sensing, and the ECM for refractive index sensing may provide an effective method for continual investigation.

## Figures and Tables

**Table 1:** Detailed values of the TMA structural parameters.

Parameter	Size (μm)	Parameter	Size (μm)
p	80	w	6
t	0.2	a	28
h	3	R	30
k	3	d	3

**Table 2:** Refractive sensing performance comparison of the suggested TMA with previous works.

Reference	Sensitivity (THz/RIU)	Q-Factor	FOM (RIU^−1^)	Resonate Frequency (THz)
[42]	1.6	225	80	4.5
[43]	1.563	100.5	-	1.628
	1.9	268.4		1.959
[44]	2	-	75	3.94
	3		50	8.28
[45]	1.1	-	-	5.9
	1.5			8.1
[46]	1.15	97.88	34.99	3.74
	2.475	216.29	76.89	7.73
This paper	2.537	234.73	147.67	4.413

## Data Availability

The data presented in this study are available on request from the corresponding author due to the restricted dissemination requirement of the corresponding project application.

## Abbreviations

TMA	Terahertz Metamaterial Absorber
RIU	Refractive Index Unit
RLC	Resistor–Inductor–Capacitor
ECM	Equivalent Circuit Model
FOM	Figure of Merit
CST	Computer Simulation Technology
